# Genome-wide identification, characterization, and functional analysis of lncRNAs in *Hevea brasiliensis*

**DOI:** 10.3389/fpls.2022.1012576

**Published:** 2022-10-05

**Authors:** Lingling Wang, Jingyi Wang, Hui Chen, Bin Hu

**Affiliations:** ^1^ Ministry of Education Key Laboratory for Ecology of Tropical Islands, Key Laboratory of Tropical Animal and Plant Ecology of Hainan Province, College of Life Sciences, Hainan Normal University, Haikou, China; ^2^ Rubber Research Institute, Chinese Academy of Tropical Agricultural Sciences, Haikou, China

**Keywords:** long non-coding RNA, tissue specificity, rubber, RNA sequencing, biological function

## Abstract

Natural rubber (NR) is an essential industrial raw material widely used in our life. *Hevea brasiliensis* (Reyan7-33-97) is an economic plant producing natural rubber. Long non-coding RNAs (lncRNAs) are emerging as crucial regulators in numerous biological processes while the characterization and analysis of lncRNAs in *Hevea brasiliensis* are still largely unrevealed. We integrated the transcriptome datasets from multiple tissues to identify rubber lncRNAs. As a result, 12,029 lncRNAs were found and characterized with notably distinctive features such as longer exon, lower expression levels and GC content, and more tissue specificity in comparison with mRNAs. We discovered thousands of tissue-specific lncRNAs in rubber root, latex, bark, leaf, flower, and seed tissues. The functional enrichment result reveals that tissue-specific lncRNAs are potentially referred to particular functions of tissues, while the non-tissue specific is related to the translation and metabolic processes. In the present study, a comprehensive lncRNA dataset was identified and its functional profile in *Hevea brasiliensis* was explored, which provides an annotation resource and important clues to understand the biological functions of lncRNAs in *Hevea brasiliensis*.

## Introduction

The eukaryotic genomes are pervasively transcribed (>90%). Except for the protein-coding RNAs (approximately 2%), a myriad of transcripts referred to as non-coding RNAs (ncRNAs) (Chekanova et al., 2007; Kapranov et al., 2007), including long non-coding RNAs (lncRNAs) that are >200 nt in length, possess little or no discernable protein-coding ability, have been reported to be involved in regulating various biological processes (Rinn and Chang, 2012; Jin et al., 2013; Zhang et al., 2014). Based on their genomic localization relationship with the protein-coding genes, lncRNAs can be classified as intronic lncRNAs, antisense lncRNAs, intergenic lncRNAs (lincRNAs), etc. (Derrien et al., 2012). Differing from protein-coding genes, most lncRNAs share poor sequence conservation between species, present relatively low but tissue-specific expression patterns, raising their special functions in regulating tissue development, cellular and biological processes.

Animal lncRNAs have been proved to be implicated in modifying chromatin, regulating transcription, posttranscription, and other cellular processes (Geisler and Coller, 2013; Cech and Steitz, 2014). In the past decades, the genome-wide identification of plant lncRNAs is later and less comprehensive when compared with mammalians. While, with the rapid advancement of sequencing technology and bioinformatics analysis, thousands of lncRNAs have been identified and the functions of some lncRNAs have been demonstrated in several plant species. In *Arabidopsis*, more than 6000 lncRNAs have been identified, classified, and annotated under normal or stress conditions (Zhao et al., 2018). Among them, a lncRNA named as *MAS* is induced by cold, activates the transcription of *MAF4* and suppresses the precocious flowering (Zhao et al., 2018). In addition, lncRNAs have been reported to function in light response (Wang et al., 2014), stress responses (Di et al., 2014; Qin et al., 2017), seedling greening modulation (Wang et al., 2018b), and transcriptional modulation processes (Rigo et al., 2020). In rice, Wang et al. found that overexpression of *LAIR* (*LRK* Antisense Intergenic RNA) confers transgenic rice increased grain yield by upregulating the expression of neighboring *LRK* genes (Wang et al., 2018c). Additionally, 3488 high-confidence maize lncRNAs were identified under drought stress, and 1535 of them were drought responsive (Pang et al., 2019). Besides, several lncRNAs were identified to be mediated in pathogen/fungal stress responses/defenses in Grapevine, wheat, rubber tree, and other plants (Zhang et al., 2013; Xing et al., 2019; Yin et al., 2019; Zhang et al., 2020; Liu et al., 2022). Although some lncRNAs have been reported in the rubber tree, the previous study is solely conducted in a single tissue, leading to a limited lncRNAs in discovery (Yin et al., 2019; Li et al., 2021; Liu et al., 2022). Thus, the identification, characterization, and functional profile of rubber tree lncRNAs were still in its infancy.

The Para rubber tree, *Hevea brasiliensis*, is a kind of economically significant tropical tree that cultivated in South America, Malaysia, Indonesia, Thailand, and China. In China, *Hevea* Reyan7-33-97 was one of the elite cultivars (Tang et al., 2016). And the natural rubber (*cis*-1,4-polyisoprene) produced by *H. brasiliensis* accounts for >98% of the natural rubber production, which is a high-quality and indispensable source for numerous rubber products worldwide (van Beilen and Poirier, 2007). In the present study, we identified 12,029 lncRNAs from multiple rubber tissues. Following, we found that the rubber lncRNAs exhibited notably distinctive features when compared with mRNAs, such as longer exon, lower expression, lower GC content, and higher tissue-specificity. Furthermore, thousands of tissue-specific lncRNAs in six tissues were identified depending on the tissue-specific score, revealing their potentially particular functions in tissue development and tissue-associated biological processes. The above results provide important clues to understand the functions of rubber lncRNAs and will lay a theoretical underpinning for further functional studies of lncRNA in *Hevea brasiliensis* and other plants.

## Materials and methods

### Transcriptome assembly

We obtained the RNA-seq data of *Hevea brasiliensis* (Reyan7-33-97) from the National Center for Biotechnology Information (NCBI) Sequence Read Archive (SRA) database (https://www.ncbi.nlm.nih.gov/sra) (**Supplementary Table 1**), and the reference genome of Reyan7-33-97 was downloaded from NCBI (https://ftp.ncbi.nlm.nih.gov/genomes/all/GCF/001/654/055/) (Tang et al., 2016; Gong et al., 2018; Wang et al., 2021). We used the fastqc (v0.11.9, http://www.bioinformatics.babraham.ac.uk/projects/fastqc/) to control the quality of RNA-seq data (Andrews, 2010) and AdapterRemoval (v2.3.1, https://github.com/MikkelSchubert/adapterremoval) to remove the adapter sequence residues from the reads (Lindgreen, 2012). After quality control, we mapped the RNA-seq data to *Hevea brasiliensis* (Reyan7-33-97) genome using hisat2 (v2.2.1, http://daehwankimlab.github.io/hisat2/) (Kim et al., 2019) and employed stringtie2 (v2.1.4, https://github.com/skovaka/stringtie2) to assemble the transcriptome of each sample. To remove the redundant transcripts, we merged the transcriptome from all samples and filtered the transcripts without strand information.

The read counts for each transcript were calculated with software salmon (v1.4.0, https://salmon.readthedocs.io/en/latest/index.html) (Patro et al., 2017). After that the Trimmed Mean of the M-values (TMM) approach was used to normalize the raw counts, and the expression values quantified as FPKM (Fragments Per Kilobase of sequence per Million mapped reads) were calculated using the edgeR (v3.30.3, https://bioconductor.org/packages/release/bioc/html/edgeR.html) (Robinson et al., 2010).

### Identification of long non-coding RNAs

**Figure 1:**
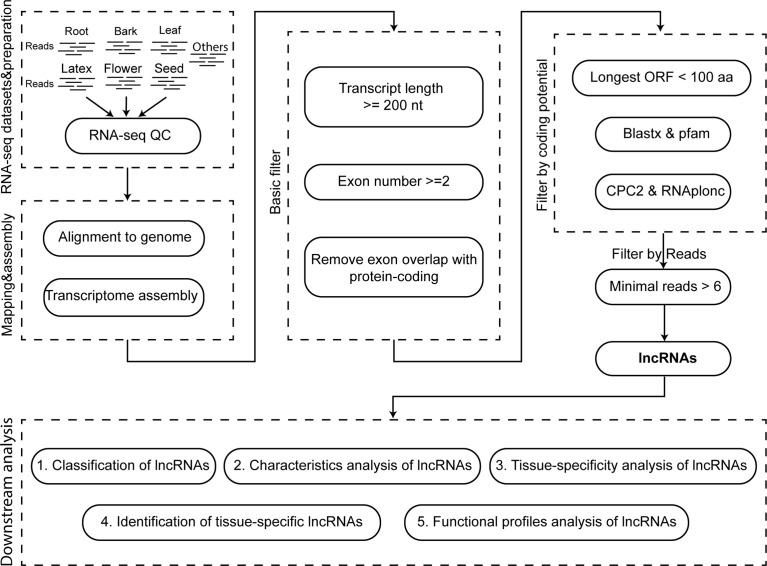
The flow chart for the identification and analysis of the long non-coding RNA. The first box indicates the RNA datasets and their quality control. The second box represents the read mapping and assemble. The third and fourth box indicate the processes for the identification and screen of the lncRNA. The fifth box is the downstream analysis of lncRNAs.

To provide a landscape of lncRNAs in *Hevea brasiliensis* (Reyan7-33-97), we integrated a stringent pipeline to identify lncRNAs (**Figure 1**). Firstly, the multi-exon transcripts >= 200 nt in length were reserved, and the transcripts, of which the exon overlapped with protein-coding genes were removed (Li et al., 2014; Zhang et al., 2014; Li et al., 2021; Liu et al., 2022). Secondly, the transcripts were filtered based on their coding potential which were evaluated by the following indicators: longest ORF length, sequence similarity or domain with the known proteins, and coding score (Yin et al., 2019; Huang et al., 2021). The TransDecoder software (https://github.com/TransDecoder/TransDecoder) was chosen to calculate the length of the longest ORF, and only the transcripts containing the longest ORF< 100 aa in length were retained. Then, we used blastx to search against non-redundant proteins (nr) of NCBI with the parameter e-value=1e-3 and employed PfamScan to search against Pfam domain with -e_seq=1e-3 & -e_dom=1e-3 (Camacho et al., 2009; Mistry et al., 2021) to filter out the transcripts share significant homology with the known protein sequences or Pfam domains. After that, to further distinguish the non-coding from coding transcripts, we used Coding Potential Calculator 2 (CPC2) (http://cpc2.cbi.pku.edu.cn/) and RNAplonc software (v1.1, https://github.com/TatianneNegri/RNAplonc) to compute their coding scores, and eliminated the coding transcripts with default criterion (Kang et al., 2017; Negri et al., 2019). Finally, to guarantee the quality of lncRNAs, the transcripts supported by at least six reads were defined as the lncRNAs in *Hevea brasiliensis* (Reyan7-33-97).

### Classification of the lncRNAs

The identified lncRNAs were classified into different groups, including intergenic lncRNAs, antisense lncRNAs, intronic lncRNAs, and other lncRNAs based on their relationships with the coding genes using FEELnc (v.0.1.1, https://github.com/tderrien/FEELnc) (Wucher et al., 2017). The intergenic lncRNAs are the lncRNAs locating in the intergenic range of coding genes, while the intronic lncRNAs are produced from intron. If a lncRNA is in the antisense strand of related coding genes, it will be defined as the antisense lncRNA.

### Characteristic investigation for long non-coding RNAs

We investigated as well as compared the transcript length, exon number, exon length, intron length, GC content, and expression values of lncRNAs and mRNAs. Here, the R (v4.0.2, https://cran.r-project.org) and R package GenomicFeatures (v1.40.1, https://bioconductor.org/packages/release/bioc/html/GenomicFeatures.html) was utilized to compute the transcript length, exon number, exon length, and intron length of the lncRNAs and mRNAs (Lawrence et al., 2013). The GC content was calculated by using the LncFinder (v1.1.5, a R package) (Han et al., 2019).

### Genome distribution of rubber lncRNAs

To examine the genome distribution of the rubber lncRNAs, we split the genome into different bins (size= 50000 nt) from the first position to the end and calculated the number of lncRNAs in each bin. The genome distribution of mRNAs was calculated with the same approach followed by comparing the genome distribution between lncRNAs and mRNAs.

### Tissue-specificity analysis

The expression values of six rubber tissues were used to compute the tissue specificity. The following formula was adopted to calculate the fraction for each lncRNA across these tissues:


$$F_{i,j}=\;\frac{E_{i,j}}{\sum_{j=1}^{n}E_{i,j}}\;\;\;\;\;n=6(n,\;Tissue\;number)$$

Here, *i* indicates the serial number of lncRNA, while *j* represents the serial number of the tissue. *F* is the fraction value; *E* represents the expression value. *E_i,j_* is the expression value of lncRNA *i* in the tissue *j*. *F_i,j_* is the fraction value of lncRNA *i* in the tissue *j*. For a given lncRNA *i*, the max fraction value among different tissues is defined as the tissue-specific score (Zhou et al., 2020). Then, the tissue-specific score of mRNAs was computed with the same formula. The tissue-specific score > 0.6 was set as the cutoff to screen the tissue-specific lncRNAs among all tested tissues.

### Biological function analysis for lncRNAs

Functional analysis of the identified lncRNAs was conducted depending on the functional annotation of their co-expression mRNAs (Bhatia et al., 2020). To find the co-expression mRNAs for lncRNAs, we performed the Pearson correlation analysis of them using R package, stats (v4.0.2, http://www.R-project.org/) with the FPKM value, and the screen criteria was set as: Pearson correlation > 0.8 & adjusted *P*-value< 0.05. Then, we performed the Gene Ontology (biological process category) and KEGG (Kyoto Encyclopedia of Genes and Genomes) annotation for the mRNAs that co-expressed with lncRNAs using the software interproscan (v5.48-83.0, https://github.com/ebi-pf-team/interproscan) (Jones et al., 2014) and kobas (v3.0, http://kobas.cbi.pku.edu.cn/) (Bu et al., 2021), respectively. For each lncRNA, we conducted the BP (biological process) and KEGG enrichment analysis of its correlated mRNAs to predict its function. In the enrichment analysis, the adjusted *P*-value< 0.1 was set as the cutoff to determine the significance of enrichment, and the *P*-value was calculated by Hypergeometric test and adjusted by Benjamini & Hochberg.

## Results

### Thousands of long non-coding RNAs were identified in *Hevea brasiliensis*

To build a comprehensive long non-coding RNA atlas for the Para rubber tree (*Hevea brasiliensis*, Reyan7-33-97), We collected the RNA sequencing (RNA-seq) data of root, bark, leaf, latex, flower, seed, etc. (**Supplementary Table 1**). And performed the transcriptome assembly, as well as a comprehensive analysis to identify, characterize and analyze the lncRNAs (**Figure 1**). After mapping the RNA-seq reads to the reference genome of *Hevea brasiliensis* and data assembling, we finally obtained 172,943 rubber transcripts.

**Figure 2:**
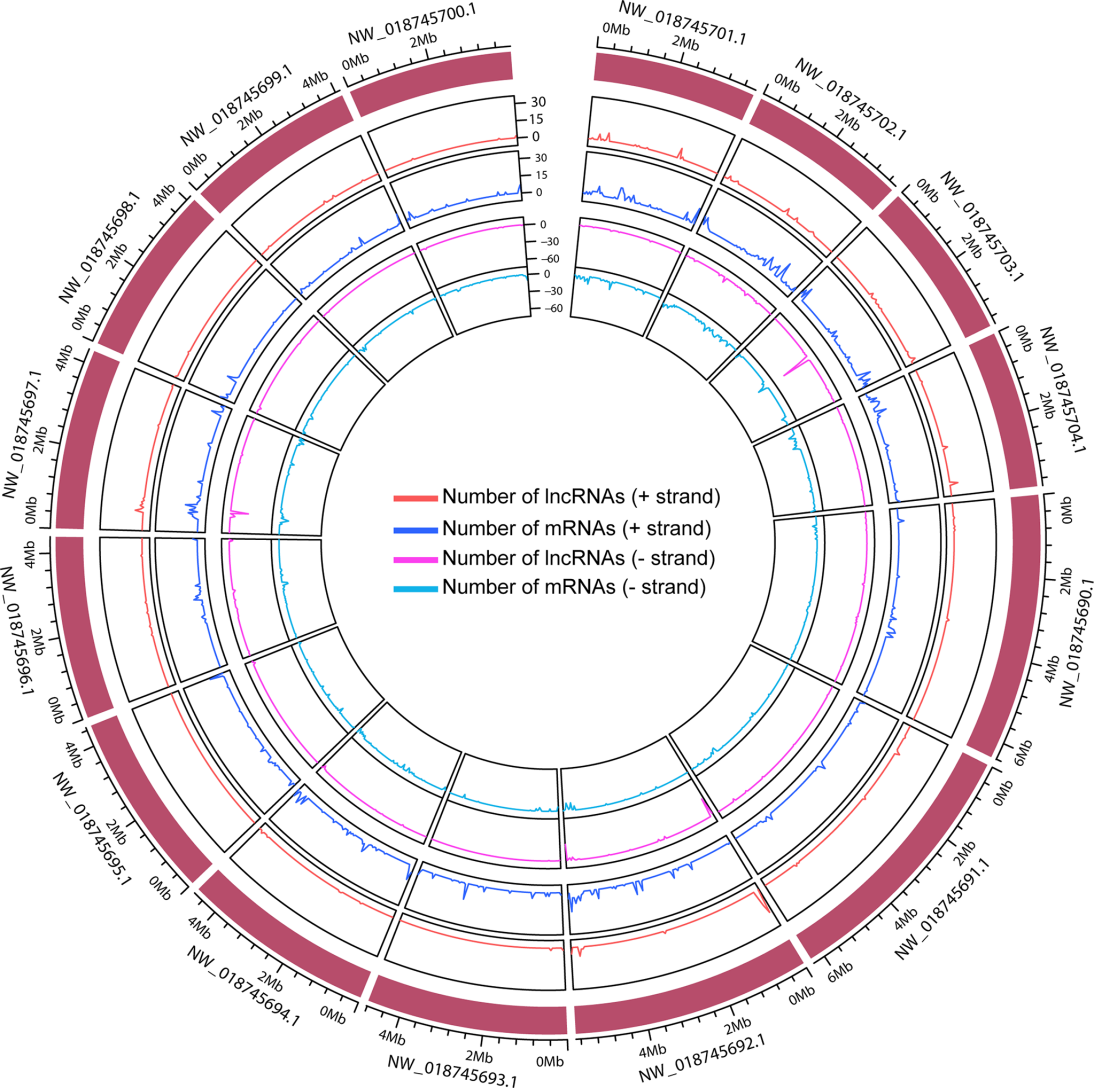
The distribution of lncRNAs and mRNAs across the top 15 length scaffolds. The out layer indicates the genomic scaffold. The orange, blue, pink, and turquoise colors represent the lncRNAs in + strand, mRNAs in + strand, lncRNA in - strand, and mRNA in - strand, respectively.

Then, we employed a stringent criterion to identify the long non-coding RNAs (detail in Materials and methods). Only the long multi-exon transcripts without the evidence of protein-coding capacity were remained, as a result, 12,029 lncRNAs of *Hevea brasiliensis* were finally identified. The lncRNAs were divided into different categories based on their genomic position relationship with coding genes, and we found that most of them were intergenic (8442, about 70%) lncRNAs, only 19% antisense lncRNAs, about 5% intronic lncRNAs and 6% of them were other lncRNAs (**Supplementary Table 2**). Moreover, we noted that although the genomic distribution of lncRNAs and mRNAs presented weak correlations in the whole rubber genome, in some of the genomic regions such as 0.4-0.8 Mb of NW_018745697.1, the number of lncRNAs exhibited high correlations with mRNAs, indicating that, to some extent, the lncRNAs presented close transcriptional relationships with mRNAs (**Figure 2** and **Supplementary Figure 1**).

### The characteristics of lncRNAs are different from mRNAs in *Hevea brasiliensis*

**Figure 3:**
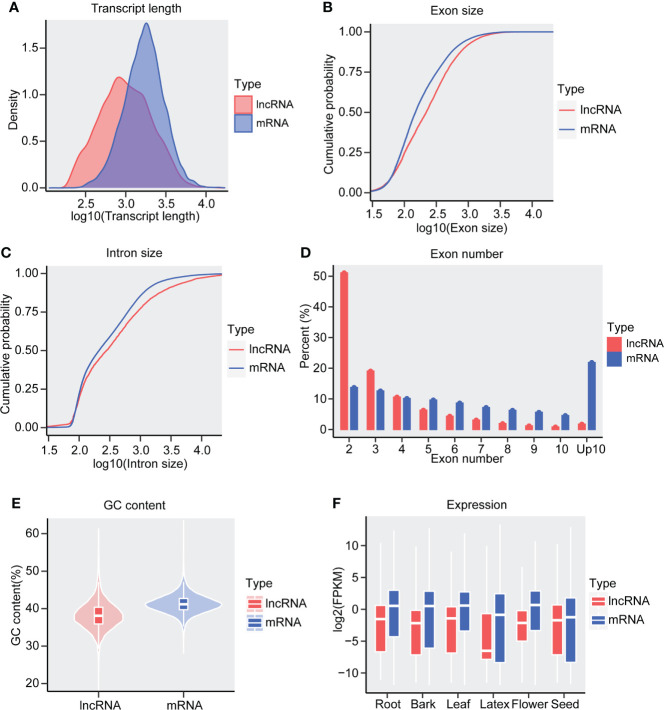
Characteristics of *Hevea brasiliensis* lncRNAs. **(A)** Density distribution of transcript length between lncRNA and mRNAs. The cumulative probability of **(B)** exon size and **(C)** intron size in lncRNAs and mRNAs. **(D)** Percent of lncRNAs and mRNAs in different exon numbers. **(E)** The GC content (%) of the lncRNAs and mRNAs. **(F)** The expression values of lncRNAs and mRNAs, Y axis indicate the expression value (log2(FPKM)).

To characterize the lncRNAs of *Hevea brasiliensis*, several features of lncRNAs and mRNAs, including exon number, transcript length, exon size, intron size, GC content, and expression values were inspected and compared. We found that the length of lncRNAs was generally shorter than that of mRNAs (**Figure 3A**), as about 80% lncRNA was 378-2668 nt in length while 80% mRNA was 805-3390 nt, which kept consistent with the features of soybean and *Camellia sinensis* lncRNAs (Lin et al., 2020; Wan et al., 2020). In addition, similarly to the lncRNAs of *Solanum lycopersicum* (Wang et al., 2018a), rubber lncRNAs exhibited longer exon and intron length than mRNAs (**Figures 3B, C**). Meanwhile, we found that only about 2% lncRNAs owned >= 10 exons in number, while more than 20% mRNA possessed more than 10 exons (**Figure 3D**), suggesting that lncRNAs possess relatively fewer exon numbers than mRNA in the *Hevea brasiliensis* genome.

The stop codons (TAG, TAA, and TGA) are preferential to harbor the A+T base and present widely distribution in the lncRNA. To examine whether the lncRNAs of *Hevea brasiliensis* have a lower G+C contents, we calculated the G+C contents of both lncRNAs and mRNAs. We noted that the rubber lncRNAs exhibit significantly lower G+C content than mRNAs (**Figure 3E**). Interestingly, we also found that the rubber lncRNAs exhibited lower expression levels than mRNA (**Figure 3F**), suggesting that the G+C content may have certain relationships with the coding potential of genes and indicating the potential of DNA transcription.

### *Hevea brasiliensis* lncRNAs exhibit higher tissue-specificity than mRNAs

**Figure 4:**
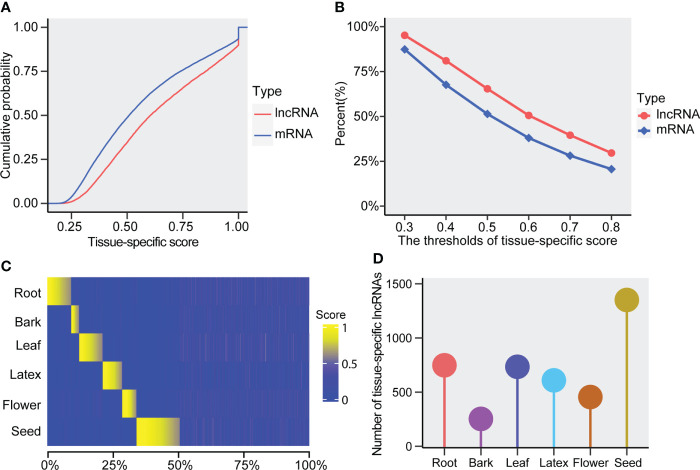
Tissue-specificity of the lncRNAs and mRNAs. **(A)** The cumulative probability of tissue-specific score in lncRNAs and mRNAs. **(B)** Percent of tissue-specific lncRNAs and mRNAs under varying thresholds of the tissue-specific score. **(C)** Heatmap of tissue-specific scores for the lncRNAs. **(D)** The number of tissue-specific lncRNAs in the tested tissues (root, bark, leaf, latex, flower, and seed).

To examine whether the rubber lncRNAs exhibit tissue specificity, we utilized the expression values of six tissues to evaluate their tissue-specific score, which was defined as the max fraction of the lncRNAs’ tissue expression value to the cumulative expressions of all tested tissues. As a result, we found the lncRNAs displayed notably higher tissue-specific scores than mRNAs (**Figure 4A**). In addition, the lncRNAs exhibited a higher percentage of tissue specificity than that of mRNAs in the *Hevea brasiliensis* (**Figure 4B**). Thus, the *Hevea brasiliensis* lncRNAs are preferred to exhibit tissue-specific expressions, suggesting that the tissue-specific lncRNAs are likely to play particular roles in the corresponding tissues. Besides, among the six tissues used in this research, we found more tissue-specific lncRNAs were identified in seed (more than 1,000), suggesting their important functions in rubber seed (**Figures 4C, D**). Meanwhile, except for bark (less than 500 lncRNAs were found), about 500-1,000 lncRNAs were respectively identified in root, leaf, flower, and latex of *Hevea brasiliensis* (**Figures 4C, D**).

### Tissue-specific lncRNAs presented several potential functions in corresponding tissues

Thousands of tissue-specific lncRNAs have been identified in *Hevea brasiliensis*, while the potential functions of them were still obscure. Gene expression is temporal and spatial, the tissue-specific expression patterns of lncRNAs also provide important clues for exploring their specific biological functions. The functional annotation of co-expressed mRNAs were used to predict the biological functions and build a functional profile for the tissue-specific lncRNAs. To obtain the co-expression relationships between these lncRNAs and mRNAs, the co-expression analysis was performed and the co-expressed mRNAs of lncRNAs were identified. Further, the Gene Ontology (GO) and KEGG enrichment analysis were conducted for the co-expressed mRNAs of each lncRNAs (**Supplementary Tables 3**, **4**).

**Figure 5:**
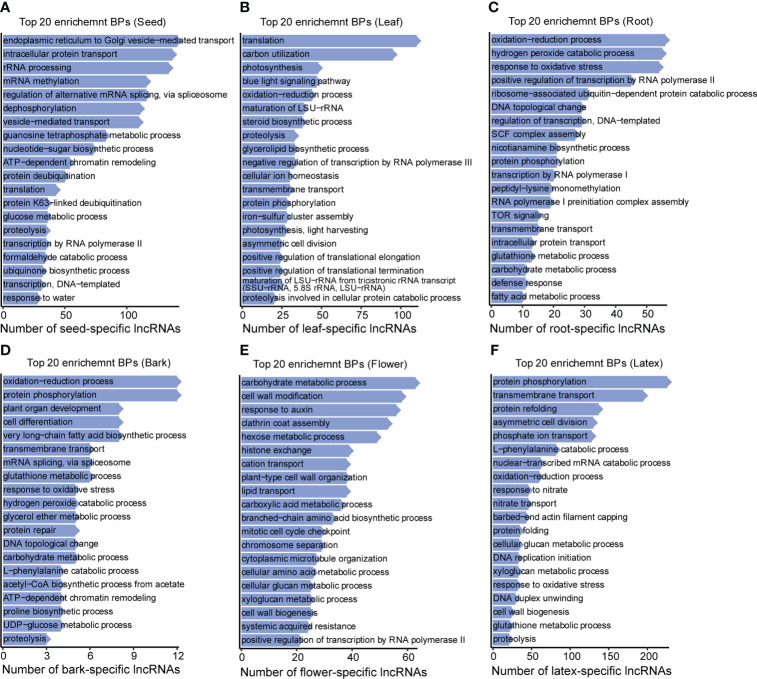
Top 20 enrichment BPs for the tissue-specific lncRNAs. Top 20 enrichment biological processes (BPs) for the tissue-specific lncRNAs in **(A)** seed, **(B)** leaf, **(C)** root, **(D)** bark, **(E)** flower, and **(F)** latex, separately. Y axis is the term of biological processes, while X axis represents the number of tissue-specific lncRNAs.

The above analyses showed that most of the seed-specific lncRNAs were enriched in the biological processes including endoplasmic reticulum to Golgi vesicle−mediated transport, intracellular protein transport, rRNA processing, mRNA methylation, mRNA splicing, transcription, translation, etc., suggesting that the seed-specific lncRNAs may be involved in transcription and protein synthesis and transport processes to prepare the storage-product accumulation for seed development (**Figure 5A** and **Supplementary Figure 2**). The leaf-specific lncRNAs were also preferentially enriched in translation, blue light signaling pathway, and photosynthesis, implying their relative activities in the protein synthesis and photosynthesis of the leaf (**Figure 5B** and **Supplementary Figure 3A**), demonstrating that the leaf-specific lncRNAs were likely to be involved in regulating the process of photosynthesis. Differently, the root-specific lncRNAs were enriched in oxidation−reduction, hydrogen peroxide catabolic, and response to oxidative stress processes, etc. (**Figure 5C** and **Supplementary Figure 3B**), indicating that these lncRNAs may function in the process of reactive oxygen species (ROS) formation (Grene, 2002). Interestingly, the bark-specific lncRNAs presented high enrichment not only in the oxidation−reduction process, but also in the protein phosphorylation, plant organ development, cell differentiation, etc. (**Figure 5D**). In the flower, the lncRNAs were enriched in the carbohydrate metabolic process, cell wall modification, response to auxin, etc., implying their crucial roles in the regulation of the above pathways (**Figure 5E** and **Supplementary Figure 4A**). Unlike the flower-specific lncRNAs, the latex-specific lncRNAs are preferentially enriched in the biological processes including protein phosphorylation, transmembrane transport, protein refolding, etc., suggesting that latex-specific lncRNAs exhibited a vital role in the protein modification and the solute transport (**Figure 5F** and **Supplementary Figure 4B**). Protein modification, as protein phosphorylation, was reported to be an important regulation form of latex biosynthetic pathway, implying the non-ignorable roles of lncRNAs in regulating latex biosynthesis. In line with GO analysis, KEGG enrichment also reveals that the tissue-specific lncRNAs are preferentially exhibited particular functions in corresponding tissues (**Supplementary Figure 5**).

**Figure 6:**
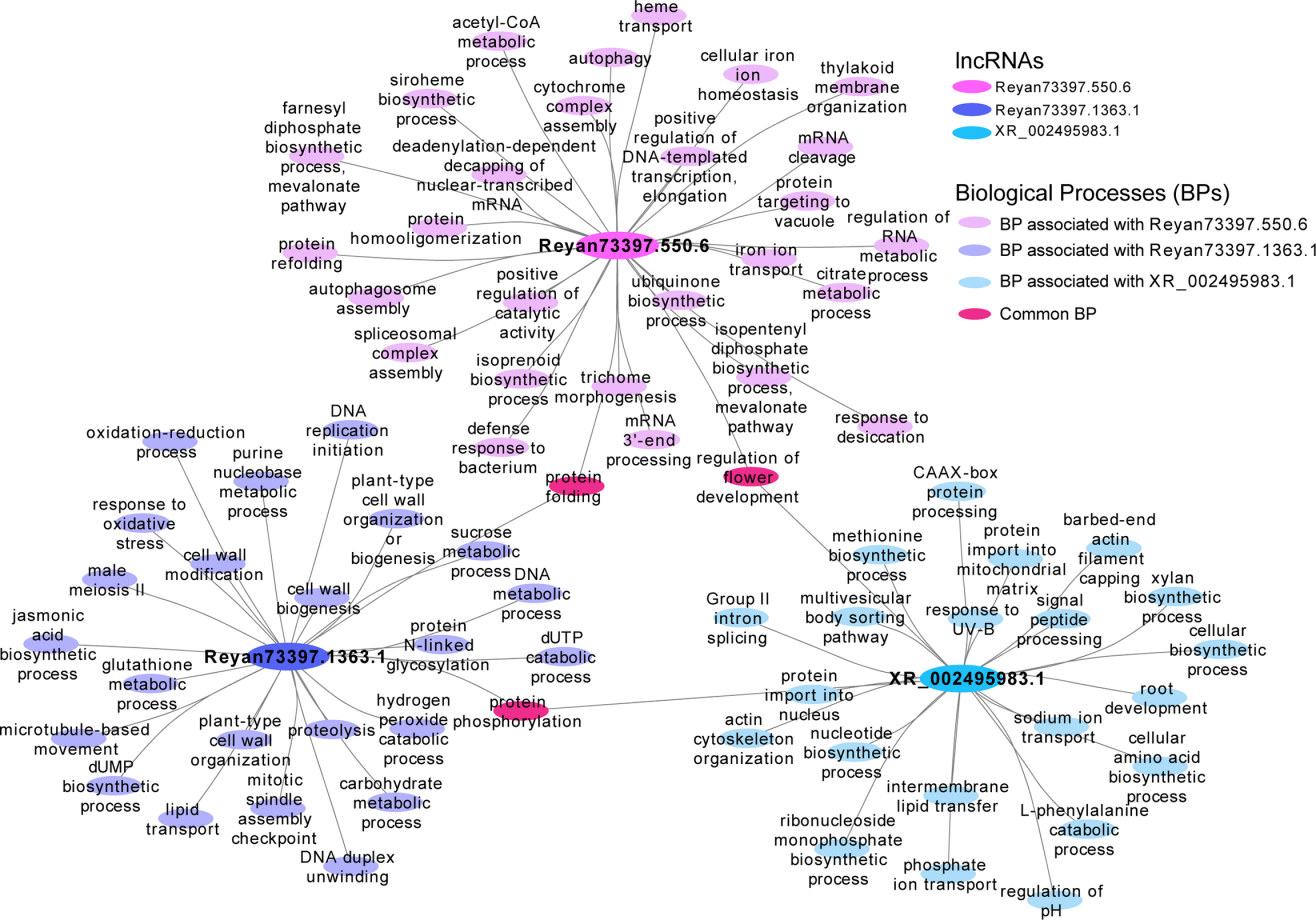
Functional network of three latex-specific lncRNAs. The biological processes in association with the latex-specific lncRNAs, Reyan73397.1363.1, Reyan73397.550.6, and XR_002495983.1. The big ellipse represents the lncRNAs, while the small ellipse indicates the biological processes.

Among the latex-specific lncRNAs, we found some lncRNAs such as Reyan73397.1363.1, Reyan73397.550.6, XR_002495983.1 are enriched in a lot of biological functions, including isoprenoid biosynthetic process, farnesyl diphosphate biosynthetic process (mevalonate pathway), isopentenyl diphosphate biosynthetic process (mevalonate pathway), jasmonic acid biosynthetic process, etc. (**Figure 6**). Meanwhile, in the latex, the KEGG enrichment of these tissue-specific lncRNAs are involved in terpenoid backbone biosynthesis, biosynthesis of secondary metabolites, and other pathways, suggesting their potential functions in the synthetic process of latex (**Supplementary Figure 5**).

### The functional profile of non-tissue-specific lncRNAs

**Figure 7:**
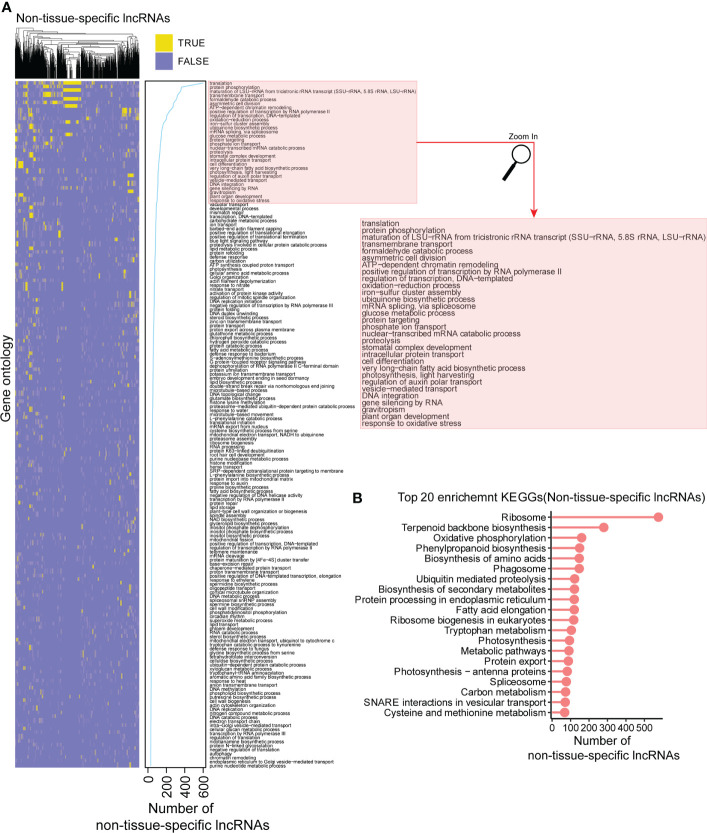
Functional profile for the non-tissue-specific lncRNAs. **(A)** The biological processes enriched in in at least 30 non-tissue-specific lncRNAs. Yellow (TRUE) indicates the significant enrichment while light blue (FALSE) represents non-significant. **(B)** Top 20 enrichment KEGG pathways for the non-tissue-specific lncRNAs. Y axis is the term of pathways, while X axis represents the number of non-tissue-specific lncRNAs.

To provide a comprehensive functional profile of lncRNAs, we also conducted a functional analysis for the non-tissue-specific lncRNAs. Among them, there were 2,181 and 1,477 lncRNAs significantly enriched in the biological processes and KEGG pathways, respectively (**Supplementary Tables 5**, **6**), and most of them were enriched in the translation, protein phosphorylation, transmembrane transport, glucose metabolic process, carbohydrate metabolic processes, etc. (**Figure 7A**). Consistently, among KEGG enrichment analysis, we found that ribosome pathway is in the top enrichment list for the non-tissue-specific lncRNAs (**Figure 7B**). The above results suggested that the non-tissue-specific lncRNAs are likely to play vital roles in the translation, transmembrane transport, and metabolic processes in multiple tissues of *Hevea brasiliensis*.

## Discussion

A growing number of lncRNAs have been reported to play important roles in various biological processes (Statello et al., 2021). Although some studies on the lncRNAs of rubber have been published (Yin et al., 2019; Li et al., 2021; Liu et al., 2022), the previously published researches mainly focused on lncRNAs in one tissue (respectively focused on the leaf, latex, and bark) rather than multiple tissues. To conduct a comprehensive identification and characterization of rubber lncRNAs, we collected and integrated the datasets from multiple tissues in the present study (**Figure 1** and **Supplementary Table 1**). As a result, we identified about 12,029 lncRNAs and found that they exhibited distinctive features when compared with the mRNAs, such as the lncRNAs possessing shorter transcript length, less exon numbers, lower expression levels and GC content, etc. (**Figure 3**), which is consistent with the previous lncRNAs reported in *Hevea brasiliensis*, maize, *Solanum lycopersicum*, and *Camellia sinensis* (Li et al., 2014; Wang et al., 2018a; Yin et al., 2019; Wan et al., 2020; Li et al., 2021; Liu et al., 2022). These results demonstrated the reliability of our results and consistency of lncRNA characteristics between rubber and other plant species. In addition, we found *Hevea brasiliensis* lncRNAs exhibited higher tissue specificity than mRNAs (**Figures 4A, B**). In this study, thousands of tissue-specific lncRNAs from six tissues were identified, more than those in previous researches (Yin et al., 2019; Li et al., 2021; Liu et al., 2022), which is mainly because of the number of tissues used for lncRNAs identification The results of functional analyses for these tissue-specific lncRNAs suggested that the tissue-specific lncRNAs could be involved in the growth and development regulation, as well as tissue-related biological processes in corresponding tissues. Which is consistent with the previous research that the lncRNAs can act in the regulation of development and stress responses, because of their significantly different expression patterns among tissues (Liu et al., 2012).

As a secondary metabolite of the rubber tree, natural rubber is synthesized from isopentenyl diphosphate which is synthesized *via* two different approaches: the plastidial 2-C-methyl-D-erythritol 4-phosphate (MEP) pathway and the cytosolic mevalonate (MVA) pathway (Tang et al., 2016). Interestingly, three latex-specific lncRNAs (Reyan73397.1363.1, Reyan73397.550.6, and XR_002495983.1) were found to be involved in the latex synthetic pathway (the mevalonate pathway). These findings will provide new perspectives to understand the particular functions of tissue-specific lncRNAs and the regulation processes of natural rubber biosynthesis. Taking together, our genome-wide identification, characterization, and functional analysis of the lncRNAs will lay a theoretical foundation for further functional studies of lncRNA in *Hevea brasiliensis* and other plants.

## Data availability statement

The datasets presented in this study can be found in online repositories. The names of the repository/repositories and accession number(s) can be found in the article/**Supplementary Material Table 1**. 

## Author contributions

LW and BH planned, designed, and wrote the manuscript, JW and HC collected the data. LW, JW, and HC analyzed the data. All authors contributed to the article and approved the submitted version.

## Funding

This work was sponsored by the State Key Laboratory of Cotton Biology Open Fund (No. CB2021A17) and the Hainan Provincial Natural Sc ience Foundation of China (No. 321QN232 and No. 322RC657).

## Conflict of interest

The authors declare that the research was conducted in the absence of any commercial or financial relationships that could be construed as a potential conflict of interest.

## Publisher’s note

All claims expressed in this article are solely those of the authors and do not necessarily represent those of their affiliated organizations, or those of the publisher, the editors and the reviewers. Any product that may be evaluated in this article, or claim that may be made by its manufacturer, is not guaranteed or endorsed by the publisher.

## Supplementary material

Supplementary Figure 1 The genomic distribution correlation between lncRNAs and mRNAs. The genomic distribution correlation between lncRNAs and mRNAs in **(A)** + strand and **(B)** - strand. One point represents one bin in the genome. The correlation calculation (R) is based on the method of Pearson.

Supplementary Figure 2 The biological processes enriched in at least 10 seed-specific lncRNAs. Yellow (TRUE) indicates the significant enrichment while light blue (FALSE) is not.

Supplementary Figure 3 The biological processes enriched in at least 10 leaf/root-specific lncRNAs. The biological processes enriched in at least 10 **(A)** leaf-specific lncRNAs or **(B)** root-specific lncRNAs. Yellow (TRUE) indicates the significant enrichment while the light blue (FALSE) is not.

Supplementary Figure 4 The biological processes enriched in at least 10 flower/latex-specific lncRNAs. The biological processes enriched in at least 10 **(A)** flower-specific lncRNAs or **(B)** latex-specific lncRNAs. Yellow (TRUE) indicates the significant enrichment while the light blue (FALSE) is not.

Supplementary Figure 5 Top 20 enrichment KEGG pathways for the tissue-specific lncRNAs. Top 20 enrichment KEGG pathways for the tissue-specific lncRNAs in **(A)** seed, **(B)** leaf, **(C)** root, **(D)** bark, **(E)** flower, and **(F)** latex, separately. Y axis is the term of KEGG pathways, while X axis represents the number of tissue-specific lncRNAs.

Supplementary Table 1 Sample information of this study.

Supplementary Table 2 The classification of long non-coding RNAs in Hevea brasiliensis.

Supplementary Table 3 The functional profile of tissue-specific lncRNAs.

Supplementary Table 4 The KEGG pathway profile of tissue-specific lncRNAs.

Supplementary Table 5 The functional profile of non-tissue-specific lncRNAs.

Supplementary Table 6 The KEGG pathway profile of non-tissue-specific lncRNAs.
