# Supplementary figures and images for: Genome-wide identification, characterization, and functional analysis of lncRNAs in Hevea brasiliensis
Source: Front Plant Sci. 2022 Oct 5;13:1012576. doi: 10.3389/fpls.2022.1012576 (PMC9581277; doi:10.3389/fpls.2022.1012576)

Figure S1

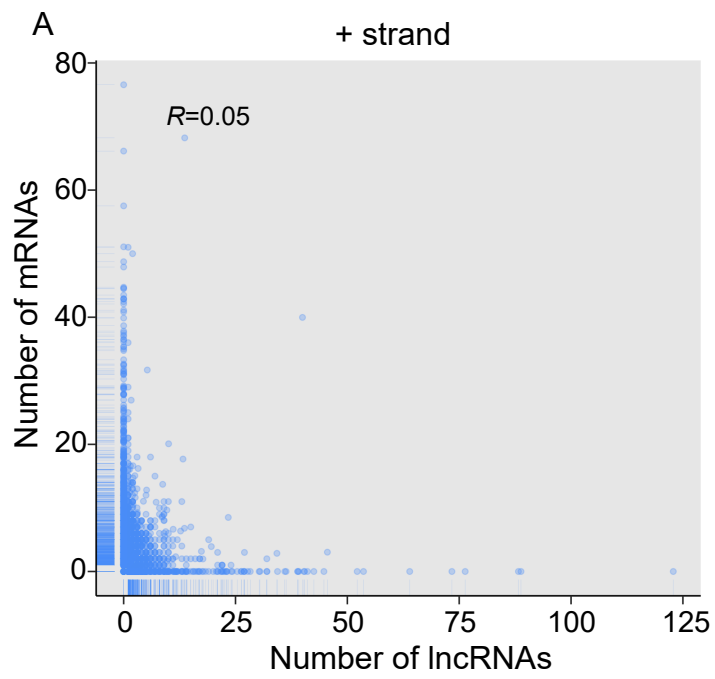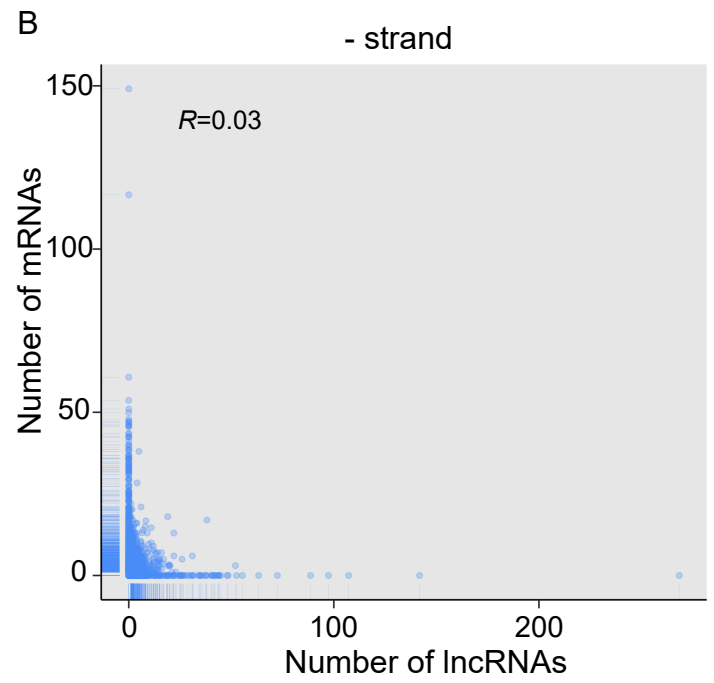

Supplement: Supplementary Figure 1 — The genomic distribution correlation between lncRNAs and mRNAs. The genomic distribution correlation between lncRNAs and mRNAs in (A) + strand and (B) - strand. One point represents one bin in the genome. The correlation calculation (R) is based on the method of Pearson. [file DataSheet_1.pdf]

Figure S2

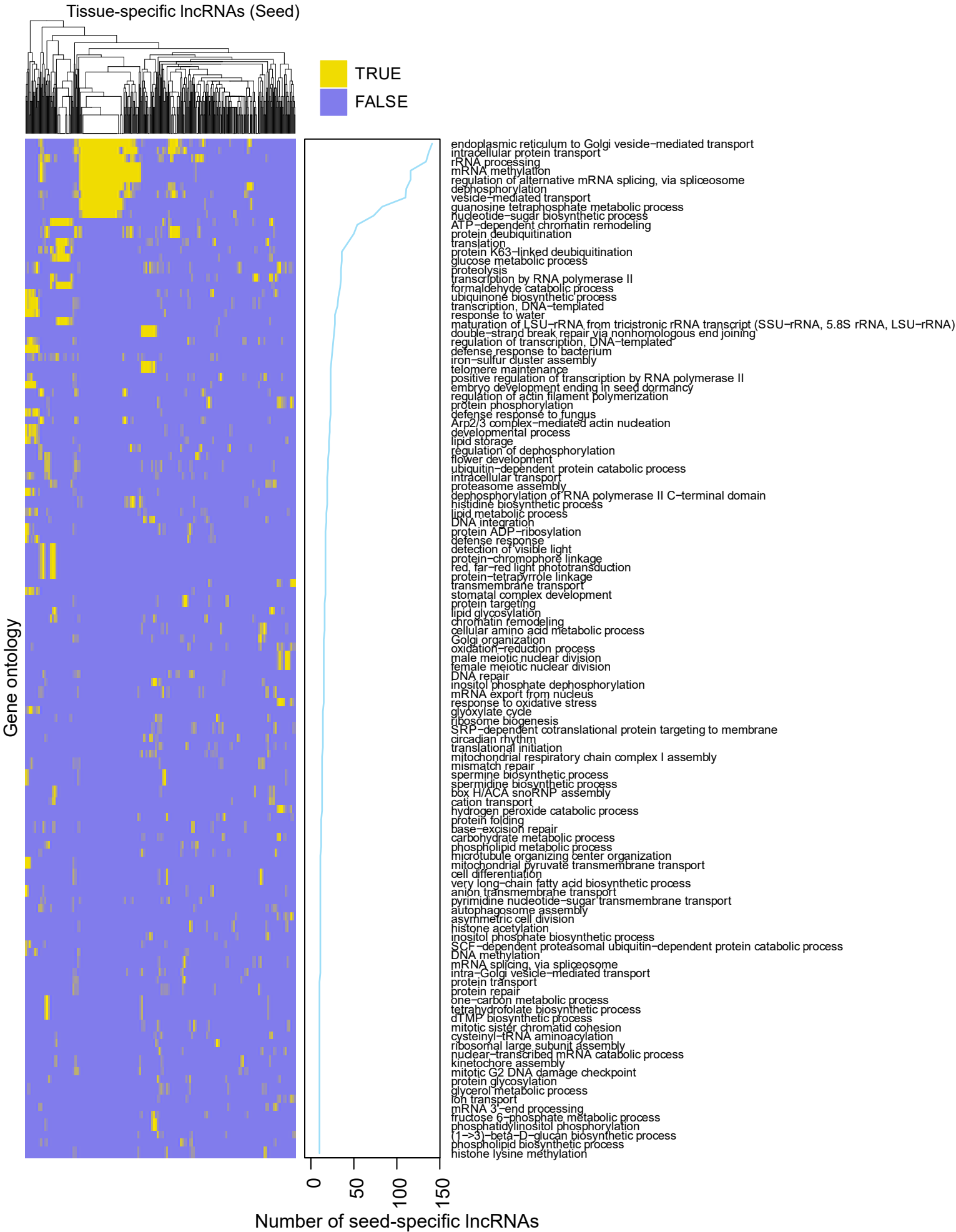

Supplement: Supplementary Figure 2 — The biological processes enriched in at least 10 seed-specific lncRNAs. Yellow (TRUE) indicates the significant enrichment while light blue (FALSE) is not. [file DataSheet_2.pdf]

Figure S3

A Tissue-specific lncRNAs (Leaf)

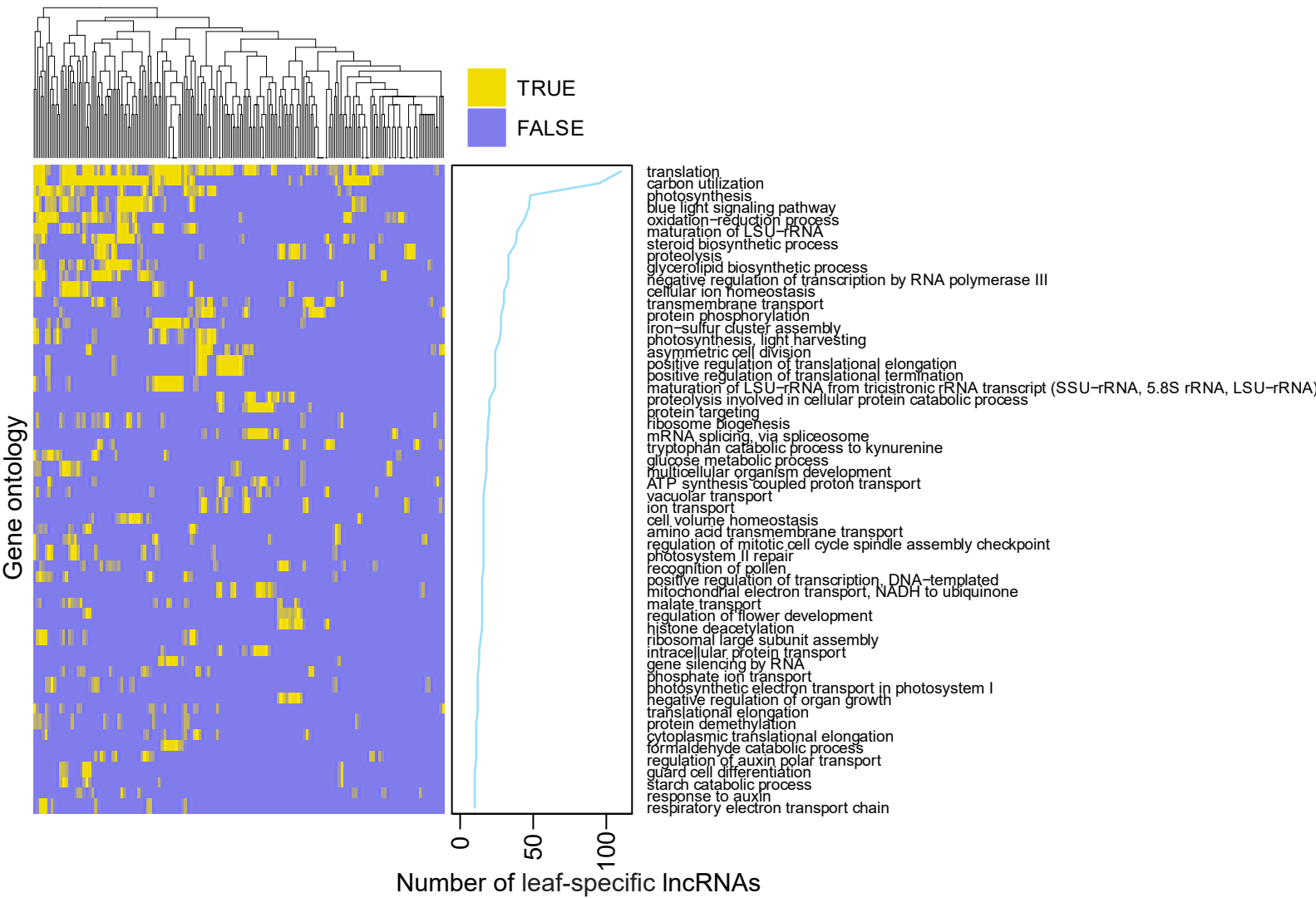

B Tissue-specific lncRNAs (Root)

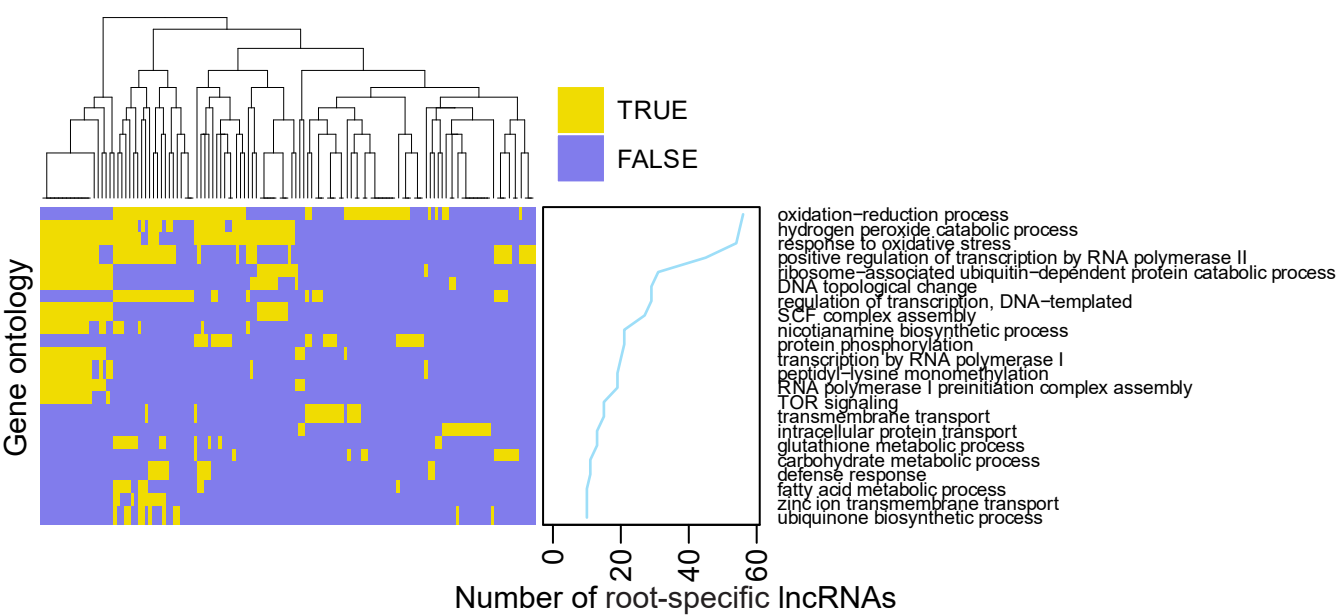

Supplement: Supplementary Figure 3 — The biological processes enriched in at least 10 leaf/root-specific lncRNAs. The biological processes enriched in at least 10 (A) leaf-specific lncRNAs or (B) root-specific lncRNAs. Yellow (TRUE) indicates the significant enrichment while the light blue (FALSE) is not. [file DataSheet_3.pdf]

Figure S4

A Tissue-specific lncRNAs (Flower)

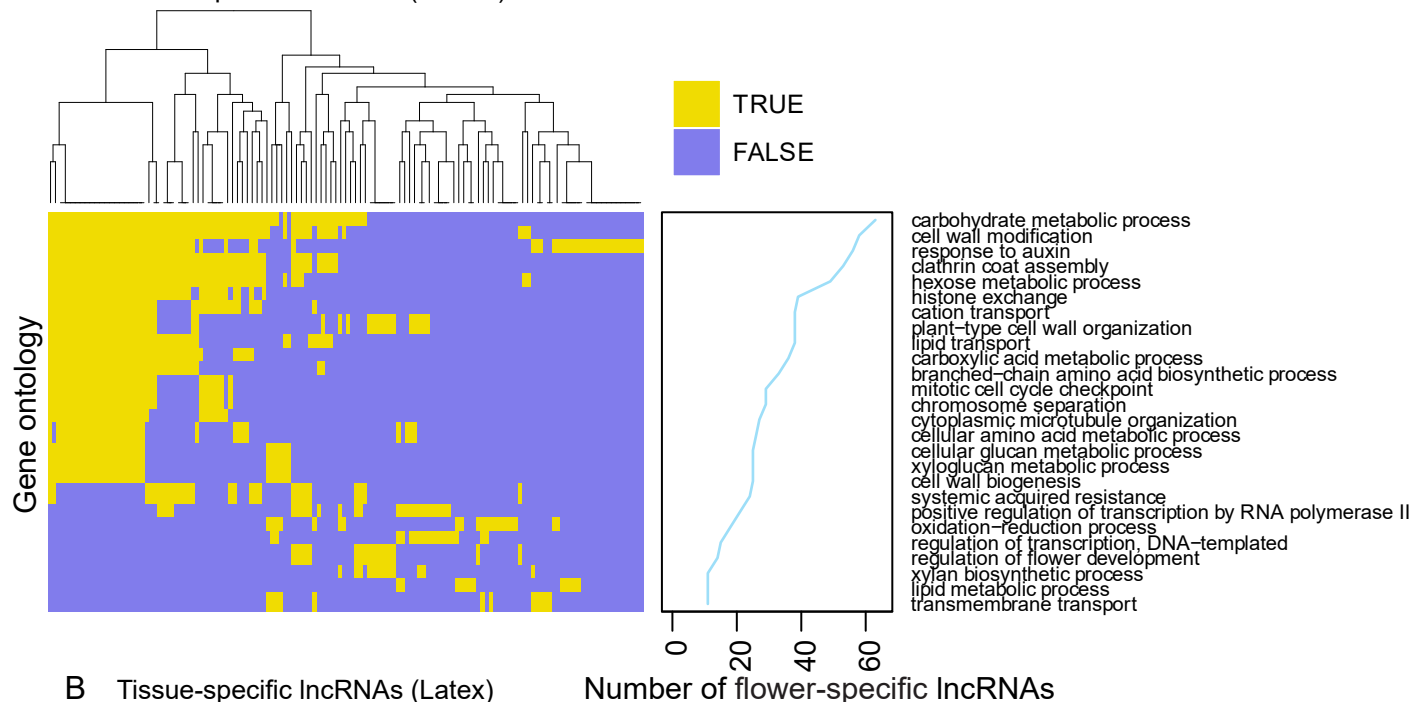

B Tissue-specific lncRNAs (Latex)

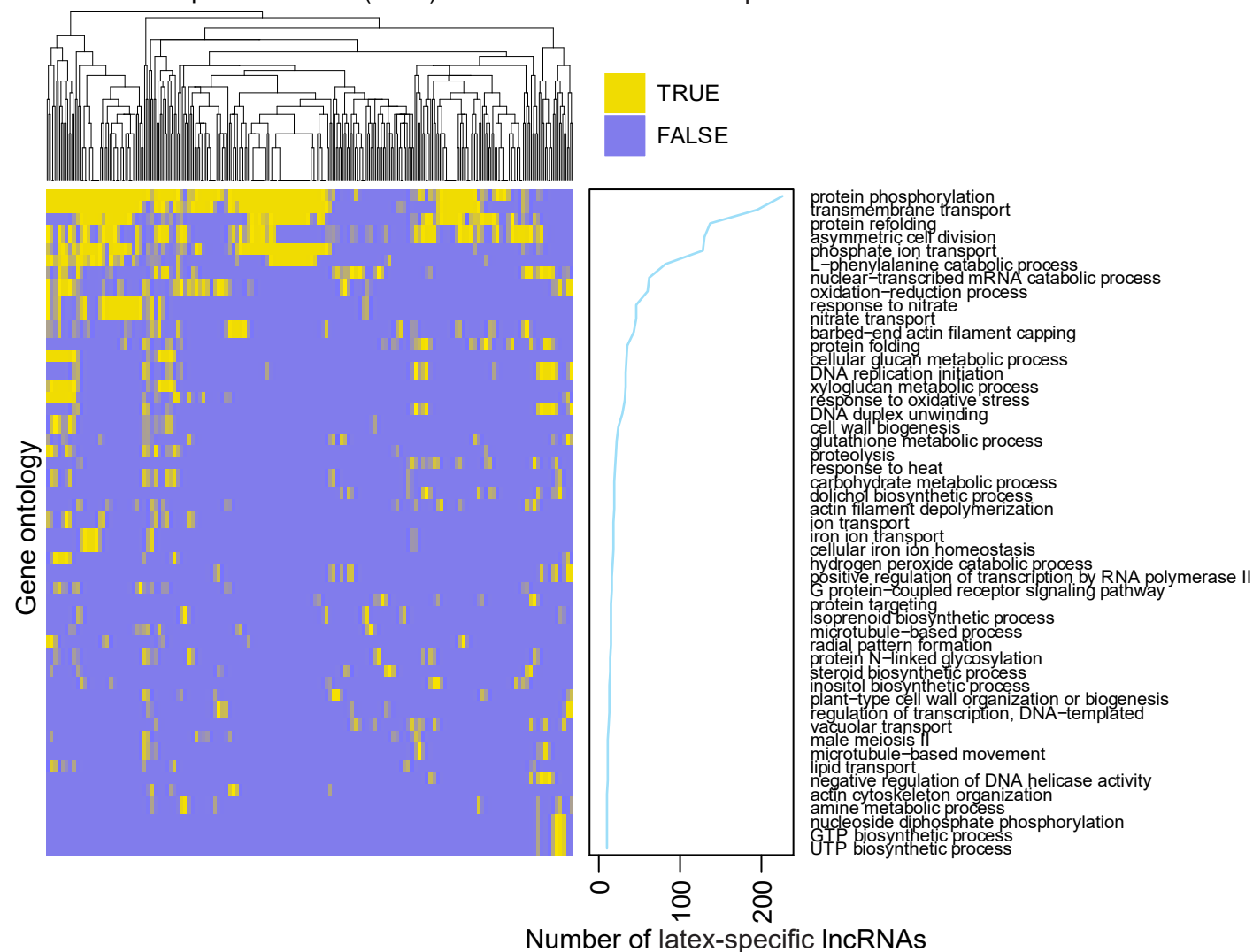

Supplement: Supplementary Figure 4 — The biological processes enriched in at least 10 flower/latex-specific lncRNAs. The biological processes enriched in at least 10 (A) flower-specific lncRNAs or (B) latex-specific lncRNAs. Yellow (TRUE) indicates the significant enrichment while the light blue (FALSE) is not. [file DataSheet_4.pdf]

Figure S5

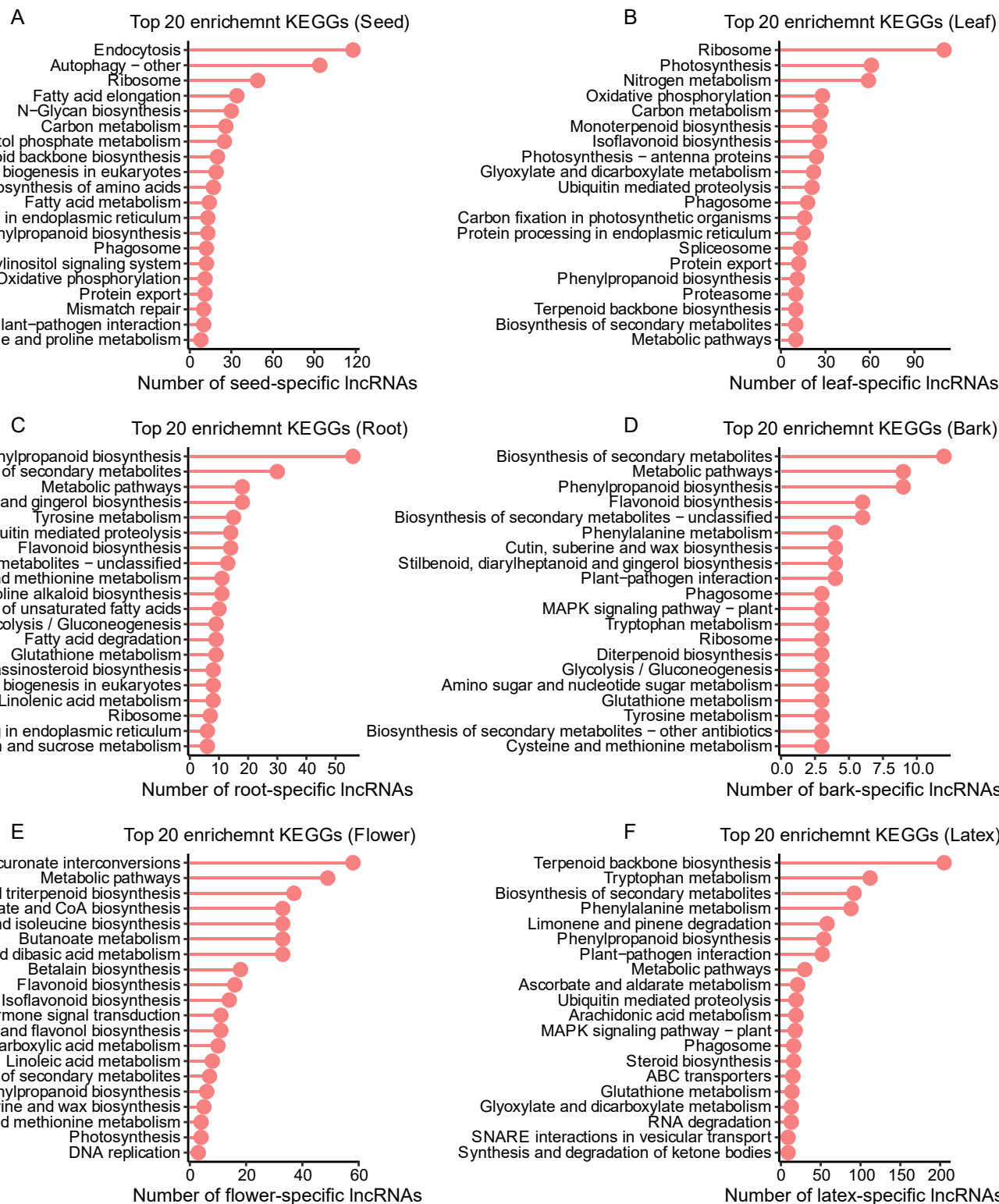

Supplement: Supplementary Figure 5 — Top 20 enrichment KEGG pathways for the tissue-specific lncRNAs. Top 20 enrichment KEGG pathways for the tissue-specific lncRNAs in (A) seed, (B) leaf, (C) root, (D) bark, (E) flower, and (F) latex, separately. Y axis is the term of KEGG pathways, while X axis represents the number of tissue-specific lncRNAs. [file DataSheet_5.pdf]
