# Supplementary material for: Genome-wide identification, characterization, and functional analysis of lncRNAs in Hevea brasiliensis
Source: Front Plant Sci. 2022 Oct 5;13:1012576. doi: 10.3389/fpls.2022.1012576 (PMC9581277; doi:10.3389/fpls.2022.1012576)
Supplement: Supplementary Table 2 — The classification of long non-coding RNAs in Hevea brasiliensis. [file Table_2.docx]

**Table S2. The classification of long non-coding RNAs in *Hevea brasiliensis***

| **Classification** | **lncRNA Number** | **Percent (%)** |
| --- | --- | --- |
| Intergenic lncRNAs | 8442 | 70 |
| Antisense lncRNAs | 2271 | 19 |
| Intronic lncRNAs | 591 | 5 |
| Other lncRNAs | 725 | 6 |
| Total | 12029 | 100 |
